# Quantifying the Child–Therapist Interaction in ASD Intervention: An Observational Coding System

**DOI:** 10.3390/brainsci11030366

**Published:** 2021-03-13

**Authors:** Giulio Bertamini, Arianna Bentenuto, Silvia Perzolli, Eleonora Paolizzi, Cesare Furlanello, Paola Venuti

**Affiliations:** 1 Laboratory of Observation, Diagnosis and Education (ODFLab), Department of Psychology and Cognitive Science, University of Trento, 38122 Trento, TN, Italy; giulio.bertamini@unitn.it (G.B.); arianna.bentenuto@unitn.it (A.B.); silvia.perzolli@unitn.it (S.P.); eleonora.paolizzi@studenti.unitn.it (E.P.); 2 Data Science for Health (DSH), Bruno Kessler Foundation (FBK), 38123 Povo, TN, Italy; 3 Hk3 Lab, 38068 Rovereto, TN, Italy; cesare.furlanello@hk3lab.ai; 4 Orobix Life, 24121 Bergamo, BG, Italy

**Keywords:** autism spectrum disorder (ASD), child–therapist interplay, observational coding system, quantitative approaches, treatment monitoring

## Abstract

Background: Observational research plays an important part in developmental research due to its noninvasiveness. However, it has been hardly applied to investigate efficacy of the child–therapist interaction in the context of naturalistic developmental behavioral interventions (NDBI). In particular, the characteristics of child–therapist interplay are thought to have a significant impact in NDBIs in children with autism spectrum disorder (ASD). Quantitative approaches may help to identify the key features of interaction during therapy and could be translated as instruments to monitor early interventions. Methods: *n* = 24 children with autism spectrum disorder (ASD) were monitored from the time of the diagnosis (T0) and after about one year of early intervention (T1). A novel observational coding system was applied to video recorded sessions of intervention to extract quantitative behavioral descriptors. We explored the coding scheme reliability together with its convergent and predictive validity. Further, we applied computational techniques to investigate changes and associations between interaction profiles and developmental outcomes. Results: Significant changes in interaction variables emerged with time, suggesting that a favorable outcome is associated with interactions characterized by increased synchrony, better therapist’s strategies to successfully engage the child and scaffold longer, more complex and engaging interchanges. Interestingly, data models linked interaction profiles, outcome measures and response trajectories. Conclusion: Current research stresses the need for process measures to understand the hows and the whys of ASD early intervention. Combining observational techniques with computational approaches may help in explaining interindividual variability. Further, it could disclose successful features of interaction associated with better response trajectories or to different ASD behavioral phenotypes that could require specific dyadic modalities.

## 1. Introduction

Studying early interactions is a core issue for research in infant typical and atypical development [1,2], and observational research represents one of its main approaches of investigation [3,4]. Developmental research is largely based on observational techniques, especially in the clinical context where ethical issues often limit the feasibility of controlled experiments [5,6]. Further, behavioral observation studies showed similar results compared to corresponding randomized controlled trials (RCTs) [7]. In line with this, researchers identified a set of quality criteria to strengthen observational results [8]. Hence, RCTs and observational studies may represent complementary approaches to balance discovery and explanation [9] increasing generalizability to the wider population [10] and generating data-driven hypotheses for subsequent confirmative designs [11]. Observational studies mainly comprise of cross-sectional, longitudinal and case-control designs [12], and have often been mistakenly considered as merely qualitative. Conversely, current observational analysis techniques allow one to collect quantitative data and to employ more sophisticated computational approaches for the systematic observation of behavior [13] This opportunity is highly relevant in the context of developmental and clinical research, where observational techniques have the great advantage of being almost completely non-invasive, or minimally invasive in many cases [3,14,15].

The study of early exchanges initially considered the dyad as a single unit with the bidirectional nature of an interacting system [16] paving the way to quantitative approaches to the study of structure and dynamics of the interplay [17]. The understanding of behavioral patterns and their flow led to the study of rhythm, reciprocity [1] and synchrony [18,19]. These aspects have mainly been investigated in the context of infant–caregiver interactions [20] with typical development, but also with respect to autism spectrum disorder (ASD) [21,22,23,24,25,26,27]; atypical neurodevelopment [28], and in at-risk clinical samples [29,30,31]. ASD refers to a set of neurodevelopmental conditions that present alteration in socio-communicative and socio-emotional domains, together with the presence of a repetitive and restrictive pattern of behaviors and interests [2]. Early core deficits mainly impact networks related to the “social brain” [32]. They disrupt early experience-dependent learning and prevent the onset of dyadic intersubjective routines that mediate child cognitive and social development [1,19,27]. Therefore, promoting adaptive social exchanges play an important part in intervention [33]. For these reasons, observational methods represent suitable approaches to identify process measures in the context of ASD intervention [34].

Several behavioral coding systems have been proposed in the literature to study interaction variables. They are broadly divided into global and micro coding scales [35]. The first group refers to general constructs and usually implies a rating judgment, often based on Likert scales, to estimate the level of specific aspects of the interaction. As an example, the construct of the emotional availability (EA) has been widely studied in the context of infant–caregiver interactions [36,37,38], and operationalized in the emotional availability scales [39], a validated research tool for parenting and clinical research [40,41,42]. Differently from global coding scales, micro-coding systems focus on behavioral manifestations with fine granularity and capture behaviors at their most specific level. Micro-coding also allows for better specificity and flexibility in the analysis. In fact, codes can be analyzed sequentially and combined, capturing the complex, interactive and time-dependent aspects of social interaction, which are considered relevant for understanding ASD [23]. However, observational methods present limitations and challenges, in particular if considered in a translational perspective. While micro-coding fine-grained schemas are easier to measure than broad constructs, they can be easily highly time consuming. Indeed, coding an interaction may require up to 5 times the length of the observation [43]. Coding at the macro level may result in being less demanding but it usually refers to general constructs with multiple dimensions that suffer from a lack of operational and quantitative definitions [3]. Moreover, the macro coding approach does not catch the structural and functional characteristics of the interaction dynamics.

For both global and micro-coding scales, coders unavoidably introduce a certain degree of subjectivity in their observations. Different metrics have been thus proposed to assess reliability and increase objectivity of the measurements, including Cohen’s k [44] and intraclass correlation coefficient (ICC, [45]). Each of these indexes has strengths and weaknesses [43,46]. Further, different forms of validity are important for observational tools in the clinical context [47] like construct (convergent and divergent) and predictive validity [48]. Moreover, longitudinal validity, i.e., the accuracy of the measure to forecast changes in variables of interest along time, may have important prognostic implications.

Of interest, micro-coding scales have the potential for advanced computational analysis. However, it is necessary to objectively and quantitatively measure interaction variables and dynamics first [13,49]. Multidisciplinary approaches based on social signal processing (SSP) can be used to acquire behavioral data from different sources [49]. Extraction of features by SSP can be automated to continuously acquire large amounts of data including verbal and nonverbal behaviors, body motion, and position tracking. Further, physiological and neural correlates [50] and interpersonal coordination can be considered [24]. Notably, machine learning techniques are increasingly applied to study interpersonal communication and social interplay [51].

As a core concept in our analysis, we considered interpersonal synchrony (IS), defined as the spontaneous rhythmic and temporal coordination of actions, emotions, thoughts and physiological processes between two or more participants [52,53,54]. When synchronized, social partners fluidly move from one state to the next [49] and show an increasing ability to cyclically flow from states of engagement and disengagement. Although these aspects were initially under-investigated [55], several works highlighted neural [56], physiological [57] and behavioral correlates of IS in human interactions [58,59,60]. Interestingly, a complex relation between IS and self-regulation has also recently been hypothesized [20,60,61,62]. Synchrony and self-regulation may be relevant in the clinical practice for interventions in the context of neurodevelopmental disorders, especially in ASD [33,63]. In the context of ASD, IS has been investigated at the biobehavioral level [50,64,65], and linked to specific social impairments [27]. However, to our knowledge, few studies investigated the role of synchrony and interaction variables in the context of early intervention [66,67,68]. The quantitative theoretical framework proposed by [58] allows for the formulation of testable hypotheses and optimal levels of synchrony may be investigated in relation to response variables and self-regulation. This approach represents a key strategy in precision medicine, where clinicians and computational systems actively cooperate to monitor, personalize and optimize ongoing treatments [69]. The interaction between clinical expertise and machine learning approaches has been recently proposed and tested with interesting results [70,71]. Therefore, an integrated perspective could provide feasible approaches to train computational models for treatment understanding, personalization and optimization. Notably, to our knowledge no observational coding scheme has been proposed in the context of child–therapist interpersonal synchrony [68].

### Aims and Hypotheses

The aim of this work is to design, propose and evaluate a quantitative observational coding system for the functional evaluation of child-therapist interactions in the context of ASD early intervention. The coding is applied first at the time of diagnosis and then after about one year of therapy. In this study, we propose a pilot design to test the coding scheme with respect to longitudinal changes in profiles of child-therapist interactions and their associations with trajectories of response to treatment. In the current study, we outline the new set of codes, the structure of the observational scheme, the coding procedures and several quantitative metrics focused on comparing the effect of therapy. Further, reliability and validity are assessed. Finally, we evaluate the coding system on a longitudinal design. Specifically, our hypotheses are: (1) the application of the coding system can detect changes in interaction profiles over time; (2) the main measure derived from the novel coding system achieves convergent validity with a widely standardized measure; and (3) the framework has predictive validity and specific dyadic aspects can be associated with outcome variables and response trajectories.

In summary, with this work we aimed at collecting preliminary evidence to support the use of the observational tool to investigate changes and monitor the ongoing process of intervention over time.

## 2. Materials and Methods

### 2.1. Participants

In this study *n* = 24 children with ASD (M = 38 months; SD = 10) were monitored over time during a developmental based intervention designed and carried out by the Laboratory of Observation Diagnosis and Education (ODFLab), a clinical research center of the Department of Psychology and Cognitive Science (DiPSCo) of the University of Trento, specialized in functional diagnosis of neurodevelopmental disorders and early interventions. Details about the model of intervention are described in Section 2.4. The families come to the Laboratory for a diagnosis and a detailed functional profile of the child. Afterwards, they undertake a personalized intervention considering the peculiar functioning of the child. At first, families are informed with respect to the research projects in which they could be involved. If they agree, they sign the informed consent to use the anonymized clinical data and video-recordings after being adequately informed about the procedure. The sample was recruited from a clinical population of *n* = 60 children assessed at ODFLab in the age range from 18 to 60 months. Inclusion criteria for this study comprised (1) a diagnosis of ASD before age 5, (2) no comorbidities with other psychiatric conditions and (3) having conducted an early intervention at ODFLab with no significant interruptions between T0 and T1, (4) having at least two assessments within 6 and 24 months, and, finally, (5) children should have a general quotient of intelligence above 40 points on the GMDS-ER. From the clinical population, *n* = 24 children met the inclusion criteria of the current study. Data were collected between 2019 and 2021.

Children went through an initial diagnostic assessment and were monitored after about one year of treatment (M = 15 months; SD = 5), with an average intensity of 2.50 (0.532) h per week. All the children were recruited at ODFLab. Diagnostic procedures applied at the clinical and research center comprise a complete functional assessment by licensed independent clinical psychologists. The nosographic diagnosis of ASD was defined following the Diagnostic and Statistical Manual of Mental Disorders—5 version criteria (DSM-5, [72]), the international clinical reference for nosographic diagnosis. We also administered the Autism Diagnostic Observation Schedule—Second edition (ADOS-2, [73]), one of the gold standard instruments for ASD diagnosis and a reference tool for research. With the ADOS-2 it is also possible to monitor treatment over time in a functional perspective, and to quantify symptoms severity [74]. Further, the Second Edition of the instrument has been developed based on the DSM-5 criteria. The diagnostic and monitoring assessments were conducted by different clinicians.

The socioeconomic status of the families, calculated with the four-factor index of social status [75] indicates a middle status for the group of participants. Demographic and general statistics are reported in Table 1.

### 2.2. Measures

#### 2.2.1. Observational Coding Scheme

**Figure 1:**
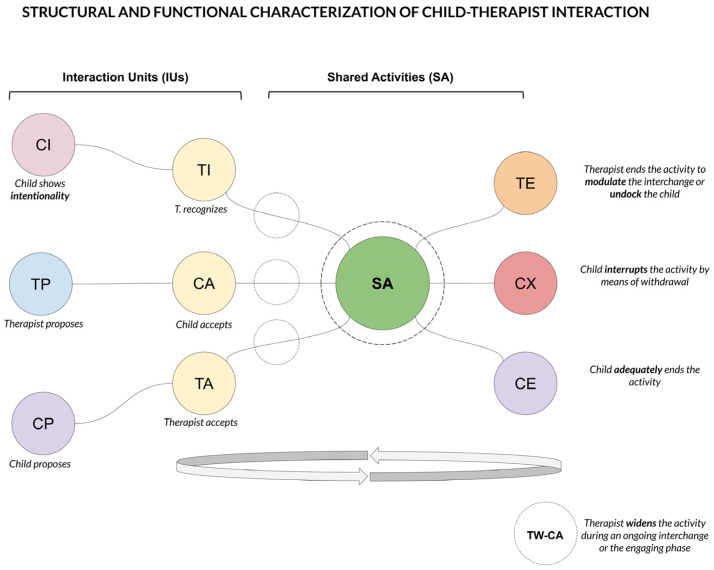
Examples of structural and functional characterization of the child–therapist interaction, described as sequences composed of interaction units leading to or leaving a shared activity state.

A quantitative observational coding system has been developed to characterize child–therapist interactions during video-recorded sessions of intervention. The code focuses on child’s intentionality and social motivation, and on therapist’s responsiveness, dyadic reciprocal adaptation and cohesion. The aim is to detect complex relationships between temporally related events characterized by multiple dimensions. The single codes refer to social units that can be multimodally coordinated and expressed by different intersubjective channels. Further, the code aims to produce data for quantitative analysis in terms of functional patterns, durations and latencies. The scheme has been designed to catch the bidirectional nature of the interchange in terms of structure and dynamics. The set of behaviors consists of 15 observational codes (13 point events and one goal state with a numeric attribute) that describe the interplay as modeled in two interconnected and recurrent scenarios: interaction units and the shared activities state (see Figure 1).

##### Interaction Units

Interaction units (IUs) provide an annotation set mainly focused on the initial phase of agreement between the social partners in terms of different types of proposals and consist of 3 couples of paired codes. From such states, child and therapist may achieve the onset of a shared activity that presents the characteristics of interpersonal synchrony. Therefore, a sequence of units can be thought of as a composition of blocks building the interaction and may be useful as a representation of both its structure and dynamics. Additionally, three codes describe the conclusion of the shared activity.

The annotations identify different types of typical social behaviors and signals mutually exchanged during the session, e.g., the therapist tries to involve the child by proposing a play or social routine (TP: therapist proposes). In turn, the child can accept (CA: child accepts), refuse (CR: child refuses) or ignore these attempts. Another interactive scenario concerns child’s intentionality (CI: child intentionality), which often represents the starting point to scaffold a play routine and it is important for the therapist to catch (TI: therapist intentionality). Therefore, the IUs assemble themselves in sequences of start and response behaviors, which are the structural patterns giving birth to the actual interplay (e.g., TP-CA, CI-TI and CP-TA). They reflect the reciprocal dynamic of the interplay in terms of engagement, involvement and agreement between the dyad through interactive patterns, which can be evaluated with specific metrics in terms of type, duration, latency and outcome. Notably, beside structure and dynamics, the coding scheme includes annotations to catch child’s emotional reactions to the degree of social stimulation, like signals of dysregulation (CD: child dysregulation) in response to therapist attempts of engagement or during intensively playful activities. Symmetrically, the ability of the therapist to recognize the child’s emotional states is coded (TR: therapist recognizes child’s state). To design the scheme, we referred to the literature in developmental research that highlight the importance of early interactions for infant development and in ASD [1,76]. As well, NDBI interventions integrate the developmental perspective in the design process of ASD early interventions focusing on the quality of the interaction, incidental learning, intrinsic motivation and shared affective states [77,78,79,80,81].

The IUs mostly represent precursors of the actual social exchange, and may or may not lead to start a social routine. If engagement results to be successful (e.g., TP-CA, CI-TI and CP-TA), the therapist scaffolds and modulates the interaction with the child, coordinating actions and sharing affective states. During these moments, called shared activities (SA) the social partners are engaged in behavioral patterns that have to be reciprocal and coordinated in order to maintain the shared experience, and thus show some degree of interpersonal synchrony. Social exchanges are characterized by an internal dynamic in which the therapist modulates the activity to promote children’s social abilities by widening the interplay (TW: therapist widens). If the child accepts (CA) and if the therapist’s widenings are proximal to the child’s abilities, the complexity of the interchange and its social demands may increase during the shared experience. In general, the exchange eventually ends by a decision of the therapist (TE: therapist ends) or after an agreement with the child to support his or her communicative intentionality. The exchange may also be unilaterally disrupted by child withdrawal (CX); clearly, measures based on these three alternatives may be relevant to monitor longitudinal changes in child’s and dyadic interactive behavior. Finally, the child can show different levels of engagement (ENG): low (1), moderate (2) and high (3). The rate is assigned based on the involvement of the child in the activity, the degree of active participation and motivation through verbal and non-verbal cues (see Table 1 for a short description of the single codes).

#### 2.2.2. Griffith Mental Development Scales—Edition Revised

The Griffith Mental Development Scales are standardized scales with satisfactory validity and reliability [82] administered to the child by trained psychologists in a laboratory setting through semi-structured activities designed to evaluate different aspects of mental development in infants and children. The instrument was standardized on an Italian population and translated [82].

They provide standardized *z* scores (M = 100; SD = 15) relative to 6 subscales in the main fundamental developmental areas that are: locomotion; personal—social; communication and listening; eye–hand coordination; performance and practical reasoning. Taken together these subscales provide a global quotient and a developmental age-equivalent that allow one to compare the scores to normalized standard with typical development. Further, specific quotients and developmental age-equivalents for each of the 6 subscales are also calculated. In this study, the learning rate [83] was computed for each child between T0 and T1 by subtracting mental age-equivalents, divided by the time elapsed between the two assessments. A LR = 1 represents a typical developmental trajectory. Learning rates represent suitable measures to monitor developmental trajectories and, in particular, whether a child is narrowing the gap between mental and chronological age (LR > 1) and could be considered as a response measure over time during intervention. Finally, LRs lower than 1 reflect an increasing developmental delay.

#### 2.2.3. Autism Diagnostic Observation Schedule—Second Edition

The Autism Diagnostic Observation Schedule-2 is a golden standard instrument for the diagnosis of ASD and its severity with high reliability and validity [73]. The administration of this tool is carried out by trained psychologists after an official ADOS-2 course. The instrument provides 5 different modules according to the child’s chronological age (from 18 months to adulthood) and expressive level of language (from no-words to fluent language). In this study, module toddler, module 1 and module 2 were used.

Each module gives a total score for the autism–autism spectrum–non spectrum classification. Further, the total score is converted into a comparison score that allows both the comparison among the modules and the classification of symptoms severity in the mild–moderate–severe range. In particular, severity levels of ADOS-2 calibrated score ranged from 3 (mild impairment in socio-communicative domains) to 9 (severe impairment in socio-communicative domains). For the purpose of this study, we considered the ADOS-2 social affect (SA) and the restricted repetitive behaviors (RRB) scores separately. The Italian standardization and translation of the instrument was used [73].

#### 2.2.4. Emotional Availability Scales

The emotional availability scales (EAS, [39]) are standardized observational scales widely used to assess the quality of emotional exchanges between parent and child. The EAS include four scales for adults (evaluating sensitivity, structuring, non-intrusiveness and non-hostility) and two for children. For the purposes of this study we focused on the two dimensions of children: responsiveness and involvement. Responsiveness refers to the child’s display of favorable signs during the interaction with the caregiver and measures how often the child responds to parents’ initiatives. Involvement refers to the child’s ability to actively involve the caregiver into the interaction through different communicative modalities (e.g., eye-contact looking, verbal involvement and body positioning). These main scales are scored from 1 to 7 on a Likert scale and they have 7 subscales each [39].

### 2.3. Early Developmental Intervention

All the participants received the early intensive intervention at ODFLab that integrates empirically validated scientific principles in a developmental frame, together with guidelines in accordance with the Italian Health System [78,79,84,85]. In order to foster intentionality and reciprocity, the aim of the therapist in this intervention protocol is to create a pleasant relationship and establish intersubjective routines by starting from the child’s own pleasure and motivation. In fact, the key aspect is providing children’s behaviors a communicative value and meaning, which is stronger when interactive situations are started from their own choice or based on shift alternation. Besides a specific work focused on restoring effective interactions, the intervention protocol aims at the acquisition of specific functional competencies through psychoeducational activities such as cognitive activities and emotional and social play. During the intervention sessions the therapist constantly monitors intervention goals, updating them on the basis of the child’s developmental improvements, and gradually increasing the complexity in terms of both cognitive loads and social requests [1,78,86].

### 2.4. Procedure

All procedures of this study were in accordance with the ethical standards of the Italian Association of Psychology (AIP), the ethical standards of the Ethics Committee of the University of Trento and the last version of the Declaration of Helsinki [87].

For each participant, 2 sessions of intervention video recorded with bird’s eye cameras were extracted, the first one after receiving the diagnosis (T0), and the second one before the final monitoring assessment (T1), for a total of *n* = 48 video files at 25 fps.

Children developmental outcomes and symptoms severity were evaluated through the GMDS and the ADOS-2. Further, available parent–infant interactions collected during the diagnostic assessment for clinical purposes were coded both at T0 and T1 with the EAS by an independent trained rater, for a total of *n* = 41 interactions. Therapists may change over time, but they followed the same protocol and objectives. Further, interventions are regularly monitored and supervised by expert clinicians. Treatment sessions were annotated by a trained observer during continuous time–event sequential micro-coding. The coding window was set to 20 min, extracted from the middle part of the session. Coders were blind to children’s developmental outcomes, symptom severity and the time point, and coding time was on average 1.5 times the length of the window. Inter-rater reliability was assessed by means of Cohen’s k [44] and two-way mixed-effects single unit with maximum agreement intraclass correlation coefficient (ICC) [45] on 20% of the videos in the sample (*n* = 10), equally distributed between T0 (*n* = 5) and T1 (*n* = 5). Cohen’s k ranged between 0.725 and 0.847 (mean = 0.767; SD = 0.037). See Table 1 for details. Video annotation was conducted using BORIS (Behavioral Observation Research Interactive Software) [88], an open source software developed by the University of Turin for behavioral observation research.

### 2.5. Data Extraction

Data extraction and aggregation were automatically performed by a pipeline of Python scripts to compute frequencies, proportions, duration, latencies and the quantitative indexes included in the analysis. The implemented indicators (descriptors) are listed in Table 2. Specific descriptors are computed to evaluate interaction sequences associated with behavioral outcomes, e.g., analyzing only the routines that ended with child’s interruption, or started with a child’s intentionality signal. Success rates can be extracted by computing the rates of response codes that are synchronous with the starting one. These variables reflect, for example, the degree by which the therapist successfully proposed activities to the child, or the degree by which they are able to catch and exploit intentionality signals to scaffold the interaction. Indicators in Table 2 applied for statistical analysis are further described in the next section.

### 2.6. Statistical Analysis

After data aggregation at the session-level and the extraction of the quantitative descriptors reported in Table 2, statistical analysis was performed within the R environment for statistical computing [89]. Data were checked for normality and homogeneity of variances through Shapiro–Wilk and Levene’s tests [90]. When appropriate, paired Welch *t* tests [91] were used to investigate longitudinal changes, as recommended in psychological research with relatively small sample sizes to reduce the probability of type I error [92]. In the presence of violations of assumptions, paired Wilcoxon signed rank with continuity correction tests [93] were performed. Effect sizes and correlations were calculated using R squared and Pearson product–moment correlation coefficient. A Bayes factor (BF) analysis was performed (package “BayesFactor”) for statistical inference [94] and interpreted according to Harold Jeffreys proposal revised by [95]. Concerning linear models, nested Bayesian regressions were performed (regressionBF) [94]. The regression models were iteratively tested against baseline models with intercept only, and against each other [94]. The selected models were checked for assumptions including homoscedasticity, normality of residuals, Cook’s distance and autocorrelation. Multicollinearity was addressed considering the variance inflation factor (VIF). With a conservative choice, the VIF threshold was set to VIF = 4 [96] and the limit for pairs of correlated predictors was set to *r* = 0.600. For backward elimination, the criterion was set to *p*-value greater or equal than 0.1 [97]. Model significance, the proportion of observed variance explained and goodness of fit were assessed with an F test, adjusted R^2^ and Bayesian information criterion (BIC) respectively [98]. As a second modeling step, repeated cross validation (CV) [99] and recursive feature elimination (RFE) procedure with random forests (RFs) were applied to the set of predictors [100,101,102] to mitigate the risk of selection bias, and performed with the package “caret” [103,104]. In particular, the 5 × 10 CV schema (5-fold CV repeated 10 times) with RF were used to estimate model metrics and predictors permutation importance metric (imp) [102]. Model accuracy metrics included root-mean-square-error (RMSE), R-squared and mean-absolute-error (MAE) [98]. The tolerance criterion to accept a more complex model over a simpler one was set at a 5% increase in RMSE accuracy.

## 3. Results

### 3.1. Demographic and General Statistics

At baseline children mental age calculated through the GMDS-ER ranged between 14 and 45 months (M = 26.500; SD = 7.223) and the language quotient ranged between 24 and 120 (M = 55.417; SD = 26.090). Further, the level of child socio-communicative impairment ranged between 6 and 19 (M = 12.333; SD = 3.319), as measured by ADOS-2 Social Affect. Further information are reported in Table 3.

### 3.2. Analytic Plan

**Figure 2:**
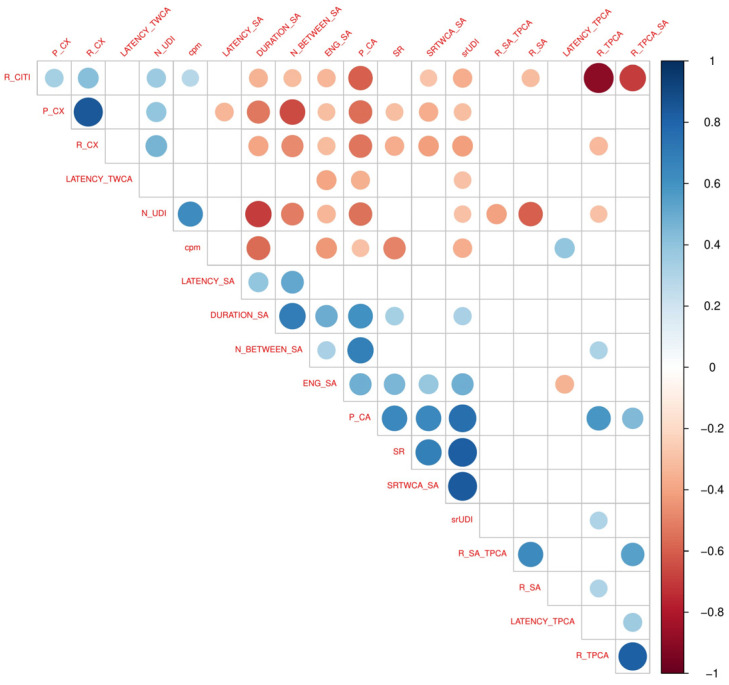
Correlation plot between behavioral descriptors (Pearson’s product–moment correlation coefficient). Significant correlations are reported (*p* < 0.05).

At first, the frequency distribution of the annotated events was analyzed at the session level. Proportional frequencies of behavioral codes at the two time points are described in Table 4. Before computing the indexes, events with insufficient numerosity or with highly sparse distribution (SD > mean) were excluded, i.e., child’s direct proposals, and dysregulation. As a second step, the set of behavioral descriptors reported in Table 2 were computed by applying the Python pipeline described in the Methods, and the correlation between the interaction indexes was explored (see Figure 2).

Absolute frequencies analysis revealed a significant (*t*(23) = 2.669; *p* = 0.014; *R*^2^ = 0.236; BF = 3.717) decrease in the total number (TOT) of event annotations between T0 (M = 94.250; SD = 27.227) and T1 (M = 74.708; SD = 22.383). Therefore, proportional frequencies were considered for the consequent analysis. Both at T0 and T1, the average proportion of child’s and therapist’s codes was between 40% and 50%, indicating a general equilibrium of behaviors within the dyad.

In order to verify our hypothesis, the analysis comprised of (1) the investigation of the longitudinal changes in the interaction features between T0 and T1; (2) the evaluation of convergent validity by computing the correlations between the behavioral indexes and the EAS subscales of responsiveness and involvement and (3) the evaluation of predictive validity by fitting data models to predict developmental and symptom severity measures starting from the observational variables.

### 3.3. Hypothesis 1: Longitudinal Changes

#### 3.3.1. Interaction Structure

Proportional frequency analysis indicated a significant (*t*(23) = −4.059; *p* < 0.001; *R*^2^ = 0.417; BF = 66.246) increase in the proportional frequency of child’s acceptance (P_CA) in response to therapist’s proposals and widenings between T0 (M = 0.240; SD = 0.061) and T1 (M = 0.299; SD = 0.064). BF analysis supports strong evidence for the alternative hypotheses. Further, it also emerged a significant (*t*(23) = 3.305; *p* = 0.003; *R*^2^ = 0.322; BF = 13.142) decrease over time in the proportion of child withdrawals (P_CX) during an interchange before (M = 0.060; SD = 0.025) and after (M = 0.039; SD = 0.026). The proportion of child’s acceptances over the total number of therapist’s proposals and widenings (SR_UDI) also showed a significant (*t*(23) = −3.788; *p* < 0.001; *R*^2^ = 0.384) increase between T0 (M = 0.635; SD = 0.114) and T1 (M = 0.767; SD = 0.141) BF analysis supported strong evidence for the alternative hypothesis (BF = 36.647). Finally, with respect to the specific IUs defining the start of interplay, the proportion of therapist’s proposals (R_TPCA) significantly (*t*(23) = −2.147; *p* = 0.046; *R*^2^ = 0.167; BF = 1.478) increased between T0 (M = 0.504; SD = 0.249) and T1 (M = 0.616; SD = 0.225).

#### 3.3.2. Interaction Dynamics

The rate of codes per minute (CPM) showed a significant (*t*(23) = 3.206; *p* = 0.004; *R*^2^ = 0.309; BF = 10.726) decrease before between T0 (M = 4.464; SD = 1.276) and T1 (M = 3.421; SD = 0.966). The total number of successful IUs (N_UDI) (i.e., code pairs that may lead to an actual interplay) showed a significant (*r* = −0.591; *t*(46) = −4.971; *p* < 0.001; BF > 100) negative association with their success rate in starting the interaction (R_SA). On the contrary, no significant (*r* = −0.12; *t*(46) = −0.866; *p* = 0.391; BF = 0.451) association emerged between the frequency of paired codes (N_UDI) and the actual rate of synchronous responses during the interaction (R_SYNC). Further, the proportion of synchronous code pairs over the total code pairs (R_SYNC) significantly increased (*t*(23) = −4.200; *p* < 0.001; *R*^2^ = 0.427) between T0 (M = 0.715; SD = 0.092) and T1 (M = 0.836; SD = 0.088), with BF analysis supporting strong evidence for the alternative hypothesis (BF = 79.093).

With respect to the actual interplay, the frequency of shared activities (N_SA) was significantly (*r* = −0.738; *t*(46) = −7.423; *p* < 0.001) associated with their mean duration (DURATION_SA) with a negative relation, i.e., the more interplays the shorter they last. Again, BF analysis indicates strong evidence for the alternative hypothesis (BF > 100). Further, the mean duration of the sharings (DURATION_SA) was significantly (*r* = 0.700; *t*(46) = 6.605; *p* < 0.001) associated also with the number of mid codes (N_BETWEEN_SA), with BF analysis indicating strong evidence for the alternative hypothesis (BF > 100). Interestingly, it also emerged a significant (*r* = −0.527; *t*(46) = −4.209; *p* < 0.001; BF > 100) negative association between the duration of the interchanges (DURATION_SA) and the proportional frequency of child withdrawals (P_CX) during the session. Further, the proportional frequency of child’s intentionality signals (P_CI) appeared to be negatively associated (*r* = −0.37; *t*(46) = −2.72; *p* = 0.009; BF = 6.826) with the durations of the interchanges (DURATION_SA).

Notably, the mean duration of the social interplays (DURATION_SA) showed a significant (V = 64; *p* = 0.013; *R*^2^ = 0.502; BF = 2.415) increase over time before (M = 144.328 s; SD = 98.640) and after (M = 233.476 s; SD = 140.148). In particular, it seems that the duration of sharings initiated by a therapist’s proposal (i.e., scaffolded and modulated from the beginning) (DURATION_SA_TPCA) increased (V = 30; *p* = 0.005; *R*^2^ = 0.607; BF = 18.576) at the end of therapy: before (M = 133.516 s; SD = 65.788) and after (M = 219.577 s; SD = 146.437). As well, the complexity of the interchange (i.e., length of sequences in SA mainly consisting in therapist’s widenings and child’s possible responses) (N_BETWEEN_SA) significantly (V = 74; *p* = 0.029; *R*^2^ = 0.443; BF = 1.857) increased between T0 (M = 5.338; SD = 4.477) and T1 (M = 8.145; SD = 6.322). Regarding interchange termination, the analysis showed a significant (*t*(23) = 2.857; *p* = 0.009; *R*^2^ = 0.262; BF = 5.336) decrease over time in the proportion of sharings ended by child withdrawal (R_CX) before (M = 0.795; SD = 0.237) and after (M = 0.584; SD = 0.277). During the sharings, the average success rate of therapist’s widenings (i.e., the proportion of widenings accepted by the child over the total number of widenings) (SRTWCA_SA) significantly increased (*t*(23) = −3.982; *p* < 0.001; R^2^ = 0.408) between T0 (M = 0.572; SD = 0.251) and T1 (M = 0.776; SD = 0.185; BF = 55.914). Finally, the average level of child engagement (ENG_SA) also significantly increased (V = 0; *p* < 0.001; *R*^2^ = 0.872; BF > 100); before (M = 1.117; SD = 0.170) and after (M = 1.694; SD = 0.297). (See Table 5)

#### 3.3.3. Longitudinal Changes in Developmental Trajectories and Symptoms Severity

During the time elapsed between the diagnostic and the monitoring assessments, the rate between mental and chronological age (rMC) significantly increased (*t*(23) = −2.862; *p* = 0.009; *R*^2^ = 0.263) over time between T0 (m = 0.718; SD = 0.199) and T1 (m = 0.782 SD = 0.276). More specifically, children showed significant gains (V = 60.500; *p* = 0.019; *R*^2^ = 0.473; BF = 7.637) in the Language quotient measured by the GMDS between T0 (M = 55.417; SD = 26.090) and T1 (M = 71.667; SD = 34.949). Further, a significant (*t*(23) = 2.828; *p* = 0.010; *R*^2^ = 0.258; BF = 5.041) decrease over time in the ADOS-2 social affect score emerged between before (M = 12.333; SD = 3.319) and after (M = 10.458; SD = 2.978). No other significant changes emerged from the analysis of cognitive functioning and symptom severity (see Table 6).

### 3.4. Hypothesis 2: Convergent Validity

To test for convergent validity, we explored correlations between behavioral indexes and child’s interaction variables, as measured by the EAS child’s involvement and responsiveness total scores. Significant correlations emerged between the latency to start the actual sharing after a successful engagement (LATENCY_SA) and both child’s involvement (*r* = 0.480; *t*(41) = 3.506; *p* = 0.001; BF = 37.826) and responsiveness (*r* = 0.411; *t*(41) = 2.884; *p* = 0.006; BF = 9.414). Decreasing involvement (*r* = −0.405; *t*(41) = −2.837; *p* = 0.007; BF = 8.534) and responsiveness (*r* = −0.380; *t*(41) = −2.632; *p* = 0.012; BF = 5.641) were associated with a higher number of IUs (N_UDI). Symmetrically, both involvement (*r* = 0.376; *t*(41) = 2.594; *p* = 0.013; BF = 5.238) and responsiveness (*r* = 0.375; *t*(41) = 2.589; *p* = 0.012; BF = 5.188) increased with the average duration of the sharings (DURATION_SA), and with the proportion of child’s acceptances (P_CA) (involvement, *r* = 0.478; *t*(41) = 3.484; *p* = 0.001; BF = 35.918) (responsiveness, *r* = 0.567; *t*(41) = 4.417; *p* < 0.001; BF = 366.633). Furthermore, child’s involvement (*r* = 0.374; *t*(41) = 2.578; *p* = 0.014; BF = 5.081) and responsiveness (*r* = 0.372; *t*(41) = 2.564; *p* = 0.014; BF = 4.936) were associated with the proportion of successful widenings during the interchanges (SRTWCA_SA) and also with the proportion of synchronous pair codes over all the pair codes (R_SYNC) (involvement, *r* = 0.346; *t*(41) = 2.365; *p* = 0.023; BF = 3.398) (responsiveness, *r* = 0.410; *t*(41) = 2.882; *p* = 0.006; BF = 9.391). Finally, involvement (*r* = 0.400; *t*(41) = 2.469; *p* = 0.018; BF = 4.122) and responsiveness (*r* = 0.445; *t*(41) = 2.178; *p* = 0.003; BF = 17.807) were associated with the average complexity see Table 6 for a synthesis) of the sharings (e.g., therapist widenings) in terms of mid codes (N_BETWEEN_SA).

Notably, significant correlations emerged for child’s responsiveness with child’s engagement (ENG_SA), (*r* = 0.356; *t*(41) = 2.443; *p* = 0.020; BF = 3.921), and inversely with proportional frequency of child’s interruptions (P_CX) (*r* = −0.385; *t*(41) = −2.673; *p* = 0.011; BF = 6.112), and with the proportion of sharings interrupted by the child (R_CX) (*r* = −0.359; *t*(41) = −2.462; *p* = 0.018; BF = 4.063).

### 3.5. Hypothesis 3: Outcome Models

The nested Bayesian regression was applied considering the most relevant interaction descriptors.

Outcome variables included (i) the rate between mental and chronological age (rMC) and (ii) the ADOS-2 social affect score (AS), for the whole sample (*n* = 48) models. Further, in order to explore predictive and longitudinal validity at the participant-level (*n* = 24), we considered (iii–iv): the learning rate at T1 estimated by behavioral indexes either at T1 or predictively at T0, respectively.

For each of the four outcomes (i–iv), the best model, i.e., the one identified by maximal BF, was selected and tested for assumptions, significance and accuracy. In case of multicollinearity (variance inflation factor: VIF > 4), the model was pruned by backwards elimination. Predictors Xi was selected starting from the highest VIF (X1), and the Pearson’s correlation table was explored. Starting from the highest correlated pair (X1,X2) of predictors, the initial predictor (X1) was removed from the model if VIF(X2) was lower or equal than 4, otherwise we dropped the feature in the pair (X1,X2) having the highest correlation with one of the other variables. The procedure was iterated until the model maximum VIF was below the safety threshold. After controlling for multicollinearity, non-significant predictors with *p*-values greater or equal to 0.1 were iteratively removed starting from the highest *p*-value.

#### 3.5.1. Predictive Validity: Relations between Developmental Outcomes, Symptoms Severity and Behavioral Descriptors

For (i), the rMC rate with *n* = 48 model is fitted by four predictors: R_CITI (rate of child’s intentionality signals over the total number of IUs); R_SA_TPCA (rate of success of therapist proposals that actually initiate the sharing); LATENCY_TWCA (latency between therapist’s widenings and child’s acceptances) and N_BETWEEN_SA (average complexity of the shared activities). The model was significant (F(4,43) = 6.390; *p* < 0.001; RSE = 0.199; max(VIF) = 1.280; adj-*R*^2^ = 0.314; BIC = −0.840; BF = 80.400). In details, the rMC model estimates are R_CITI (b = 0.587; *t*(43) = 3.670; *p* < 0.001); R_SA_TPCA (b = 0.318; *t*(43) = 3.040; *p* = 0.004); LATENCY_TWCA (b = −0.060; *t*(43) = −1.880; *p* = 0.083) and N_BETWEEN_SA (b = 0.018; *t*(43) = 3.290; *p* = 0.002). The intercept was significant (b = 0.399; *t*(43) = 3.190; *p* = 0.003). Moreover, R_CITI (im*p* = 0.727) was confirmed as the most important variable by RF importance analysis, followed by N_BETWEEN_SA (im*p* = 0.684), R_SA_TPCA (im*p* = 0.573) and LATENCY_TWCA (im*p* = 0.383).

For symptoms severity (ii), the AS (ADOS-2 social affect score) with the *N* = 48 model was fitted with the three predictors R_TPCA (rate of therapist’s proposals over the total number of IUs), ENG_SA (average child’s engagement level) and P_CA (rate of child’s acceptances to therapist’s proposals and widenings). The model was significant (F(3,44) = 9.969; *p* < 0.001; RSE = 2.600; max(VIF) = 1.96; adj-*R*^2^ = 0.364; BIC = 243.114; BF = 652.077). In details, the AS model estimates were R_TPCA (b = 7.636; *t*(44) = 9.893; *p* < 0.001); ENG_SA (b = −2.223; *t*(44) = −1.911; *p* = 0.063) and P_CA (b = −25.658; *t*(44) = −3.309; *p* = 0.002). The intercept was significant (b = 17.165; *t*(44) = 9.893; *p* < 0.001). The RF importance analysis gives ENG_SA as the most important predictor (im*p* = 173.800), followed by P_CA (im*p* = 149.500) and R_TPCA (im*p* = 121.600).

#### 3.5.2. Longitudinal Validity

(iii) The developmental learning rate (LR) estimated at T1 (*N* = 24) was fitted with the four predictors R_TPCA (rate of therapist’s proposals over the total number of IUs), R_SA_TPCA (rate of success of therapist proposals that actually initiate the sharing), LATENCY_SA (latency between child–therapist engagement and the actual beginning of the interchange) and P_CX (rate of child’s interruptions). The model was significant (F(4,19) = 8.601; *p* < 0.001; RSE = 0.331; max(VIF) = 1.271; adj-*R*^2^ = 0.569; BIC = 28.544; BF = 96.473) and explains a significant proportion of the observed variance. In details, the LR model estimates at T1 are R_TPCA (b = −0.916; *t*(19) = −2.786; *p* = 0.012); R_SA_TPCA (b = 1.088; *t*(19) = 4.505; *p* < 0.001); LATENCY_SA (b = 0.031; *t*(19) = 3.991; *p* < 0.001) and P_CX (b = 6.062; *t*(19) = 1.994; *p* = 0.061). Intercept was not significant (b = 0.244; *t*(19) = 0.751; *p* = 0.462). RF repeated cross validation highlighted R_TPCA as the most important predictor (im*p* = 1.451) followed by LATENCY_SA (im*p* = 1.264) and R_SA_TPCA (im*p* = 1.177). P_CX (im*p* = 1.031) was the least important predictor.

(iv) For longitudinal predictive validity estimated at T0, the model with *N* = 24 is fitted with the four predictors R_SA (success rate of actually starting the interplay after an IU), P_CA (rate of child’s acceptances), R_CITI (rate of child’s intentionality over the total number of IUs) and LATENCY_SA. The model was significant (F(4,19) = 3.806; *p* = 0.020; RSE = 0.414; max(VIF) = 2.248; adj-*R*^2^ = 0.328; BIC = 41.025; BF = 1.605). In details, the LR model estimates at T0 are R_SA (b = 1.738; *t*(19) = 2.923; *p* = 0.009); P_CA (b = 4.714; *t*(19) = 2.611; *p* = 0.017); R_CITI (b = 2.056; *t*(19) = 3.383; *p* = 0.003) and LATENCY_SA (b = −0.031; *t*(18) = −1.648; *p* = 0.116). Intercept was significant (b = −1.516; *t*(18) = −2.193; *p* = 0.041). RF repeated cross validation highlighted R_SA (im*p* = 1.290) and P_CA (im*p* = 1.210) as the most important predictors, followed by R_CITI (im*p* = 1.144) and LATENCY_SA (im*p* = 1.022).

The external caret analysis based on RFE confirmed three out of four models. A more conservative model was suggested for rMC (i) with only the first three predictors: (RMSE = 0.230 (0.051); *R*^2^ = 0.152 (0.145); MAE = 0.192 (0.035)). The remaining three models were confirmed: AS (ii) with three predictors (RMSE = 2.808 (0.420); *R*^2^ = 0.296 (0.177); MAE = 2.369 (0.402)); LR at T1 (iii) with four predictors (RMSE = 0.452 (0.107); *R*^2^ = 0.404 (0.299); MAE = 0.396 (0.082)) and LR at T0 (iv) with four predictors (RMSE = 0.488 (0.110); *R*^2^ = 0.271 (0.253); MAE = 0.411 (0.098)).

## 4. Discussion

The first aim of this work concerned the quantitative characterization of child–therapist interactions by means of an observational coding system during sessions of early intervention for ASD. From a general standpoint, the analysis of behavioral descriptors pointed out that the interactive patterns could be described quantitatively in a valid and reliable way. The therapist’s proposals represented more than a half (56%) of the IUs, and children’s main modality consisted of intentionality signals (36.7%). In general, the interplays due to the child’s withdrawal more than a half of the times (69%).

We explored longitudinal changes of indexes derived from the coding schema in order to explore how the schema can be used to monitor structure and dynamics of interaction along an intervention. On our data, we first noticed a significant decrease in the total frequency of behavioral annotations over time. The rate of child’s acceptances of therapist’s initiatives significantly increased over time. Further, the proportion of child’s withdrawals significantly decreased. Considering the set of IUs, the therapists’ ability to make successful proposals and widenings significantly increased over time. The schema managed to capture changes of the dyadic exchange in its structural properties during time, e.g., with an increase in the proportion of therapist’s proposals.

Considering dynamic aspects, the length of interactive pattern sequences increased along time. Further, the total number of interaction precursors (i.e., of child’s intentionality signals and child’s and therapist’s proposals) showed a negative association with their rate of success in eventually starting the interplay. Thus, a higher number of attempts actually appear to lead to less shared activity states, which is the intermediate goal of the intervention. From a clinical standpoint, this coding experiment may place the emphasis on the quality of therapist’s initiatives more than on their quantity. Notably, we measured a significant increase in the proportion of synchronous pair codes (i.e., events followed by the appropriate response) at the end of the intervention. The coding system thus identified a tendency towards a greater level of dyadic synchronization and attunement.

Focusing on shared activities, the analysis again showed that a greater number of shared events were associated with shorter exchanges. Further, longer interplays were more likely to be interrupted by the child. On the contrary, sharings with greater durations had greater complexities in terms of widenings of the activity. At the end of the intervention, dyadic interactions tended to be longer, more complex and less frequently interrupted by the child. Dyads also improved attunement during more elaborated activities. In summary, the coding schema led to finding substantial evidence for an increasing proportion of dyadic exchanges terminated by more socially adaptive and functional strategies rather than child’s unilateral interruption or disengagement. Therefore, our data seem to support the hypothesis that the code is able to detect changes in interaction variables over time.

Our second hypothesis concerned the convergent validity of the coding system. Considering the EAS child’s scales of responsiveness and involvement, both subscales were correlated with the total amount of dyadic interactive attempts and with the proportion of child’s acceptance. In addition, higher scores in the subscales were related to increasing latency to start the interchange, duration and complexity. Further, higher scores in responsiveness and involvement were associated with a higher proportion of synchronous behaviors and more effective therapist’s widenings. Therefore, coherently with the hypotheses, these global dimensions of child’s interactive behaviors measured by the EAS can be studied in terms of dyadic characteristics described by quantitative analysis of the interplay, possibly leading to identify specific interaction patterns associated with the better child’s emotional availability.

Notably, the child’s engagement level and the proportion of child’s interruptions correlated with child’s responsiveness. More responsive children showed better abilities to modulate the interchange in terms of communicative intentionality, and to conclude the interplay without interruptions. The correlations emerged from our data give some first indications about the observational coding scheme convergent validity.

Third, we tested for predictive validity of the new coding schema considering outcome variables associated to developmental trajectories and symptom severity, Developmental models of intervention represent a key strategy for early ASD treatment validated by a growing amount of research [105,106,107,108]. These approaches stress the role of interaction variables, shared pleasure, child engagement and motivation as active ingredients of intervention [108,109].

In particular, in our first model the gap between mental and chronological age was predicted by (1) greater proportion of child’s intentionality signals, (2) more effective therapist’s proposals to actually initiate the interchanges (3) greater complexity of the interchanges and (4) faster child’s responses to therapist’s widenings. These results are coherent with literature reporting different response trajectories during intervention in terms of developmental outcomes, also suggesting as a next analysis step the stratification into clusters of trends, which once understood in terms of quantifiable features may help designing more impactful intervention strategies [14,15,83,110].

In the second model, higher levels of social impairment (ADOS-2 social affect score) were predicted by (1) greater proportion of therapist’s proposal, (2) lower levels of child’s engagement and (3) lower predisposition of the child to take part in the exchange and get involved by therapist’s attempts.

These results link interaction variables to both cognitive and social aspects. Interactive behaviors associated with less impaired developmental profiles include better therapist’s abilities to involve the child and scaffold more elaborated exchanges. Further, the child shows better intentionality. Instead, social difficulties appeared to be related to lower levels of engagement, less intentionality and responsiveness. Interestingly, the role of engagement has been recently highlighted by literature on ASD early treatment response [68].

Our third and fourth models considered the developmental learning rate over time, to explore the association between interaction variables and different response trajectories post- and pre-intervention. Better response trajectories were predicted by (1) lower proportions of therapist’s proposals, (2) more successful therapist’s proposals, (3) longer delays to initiate the interchange and (4) higher proportions of shared activities interrupted by the child.

Finally, better outcomes were predicted at the time of diagnosis by interactions having (1) more successful therapist’s proposals, (2) higher levels of child’s intentionality, (3) better child’s abilities to accept the other’s initiatives and (4) shorter time to actually start the exchange.

The quantification induced by the coding schema thus is expected to highlight child’s intentionality as a key variable in determining interaction structure and dynamics, and the role of therapists’ abilities to find the most effective strategies to (i) successfully involve the child and (ii) optimally scaffolding and regulating the interaction. In fact, the role of the child’s acceptances and interruptions appear to be particularly interesting and need to be better understood. While interacting with a child who is more capable to accept and respond to the other’s social initiative may represent a good marker at the beginning of the intervention, better response profiles are also predicted by an increased proportion of child’s interruptions over time. Considering that longer interactions are associated with greater complexity and that their duration increases over time, this may reflect increasing social demands introduced by the therapist during play. In this perspective, interruptions followed by a further high-quality interaction may reflect an interplay that develops in the “zone of proximal development” [111]. In turn, this may require higher self-regulation strategies, thus increasing the risk for the child that the social stimulation exceeds his regulatory abilities, eventually leading to more interruptions. Interestingly, recent evidence highlighted a complex relationship between interpersonal synchrony and self-regulation [58,60] and may also be relevant for intervention [33,66,67,68]. To conclude, data models are in line with our third hypothesis. In fact, results seem to support the coding scheme predictive validity and its validity to link interaction and outcome variables.

## 5. Limitations

This study presents some limitations. First, the sample is relatively small. Hence, beyond the validity of the coding schema, these observational results should be considered as preliminary indications to be further investigated. Secondly, two time points restrict the possibility to perform more robust analyses, especially concerning the capability of the schema to identify clusters of response trajectories. In fact, additional intermediate time points are needed to enable a time series analysis, increasing longitudinal predictive validity and helping in disentangling different response paths that may be associated with specific interactive patterns. We expect that more midpoints in terms of video recorded sessions allow one to unravel dyad stratification patterns in potentially high dimensional spaces, and also to explore the existence of sensible timepoints for treatment progress. In general, therapists can change over time. These shifts are often motivated by clinical considerations, e.g., to provide the child the opportunity to interact with different social partners to increase flexibility and generalize competencies, and by practical reasons. Clearly, therapists’ alternation makes the analysis more complex but can be used to disentangle therapists’ and children’s role in the dyadic exchange. Finally, the lack of a comparison group limits the possibility to better understand changes related to the intervention.

## 6. Conclusions

In this work we introduced and evaluated a novel quantitative observational approach based on a micro-coding of child–therapist interplay in terms of interaction units and shared activities states. The approach is specifically designed to support the study of the child–therapist interaction in the context of early ASD intervention, also opening the way to applying computational methods for analysis and predictive modeling. Beyond providing construct validity of the coding, on our data we found preliminary indication of significant changes of dyadic interactive elements at the end of the intervention. Notably, the method has a potential for developing predictive models of outcome based on child–therapist interchange patterns. We expect that the application of the method to midpoints in the intervention may help stratifying into subgroups, defined by a combination of therapist attunement and child behavioral phenotypes that could influence interactive, intersubjective and social modalities. Differences in developmental and symptom severity trends and their impact on treatment response already emerged [83,112]. In fact, optimal strategies to engage the child during may depend, at least partly, on such ground interaction features.

From a theoretical perspective, objectively studying dyadic aspects may enhance knowledge of both the interaction structure and dynamics of the interplay. In fact, research emphasizes the relevance of understanding the “hows” and the “whys” of treatment response [113]. However, literature largely focused on child’s outcomes, while interaction variables still remain under-investigated mainly due to the lack of quantitative measures and tailored instruments [86].

From a clinical standpoint, linking child–therapist behavioral patterns with developmental outcomes, symptoms severity and response trajectories may represent an initial and promising effort to design optimized and personalized interventions. In a translational perspective it may inform clinicians with therapeutic feedback and monitoring tools with a precision approach [14]. Unraveling interaction variables may enlighten their role in response trajectories and help in explaining interindividual variability [15,110], and in bridging research to clinical practice [68,74,79].

## Figures and Tables

**Table 1:** Observational coding system and intraclass correlation coefficient (ICC).

Code	Description	ICC (Alternative Hypothesis r0 > 0.8)
TP *	Therapist proposes	0.958; F(9,5.100) = 5.400; *p* = 0.038 (0.764–0.990)
TW *	Therapist widens	0.953; F(9,9.090) = 4.620; *p* = 0.016 (0.824–0.988)
CA *	Child accepts	0.952; F(9,5.580) = 4.600; *p* = 0.039 (0.765–0.989)
CR	Child refuses	0.627; F(9,9.49) = 0.477; *p* = 0.858 (0.096–0.889)
CI *	Child’s signal of intentionality	0.940; F(9,7.990) = 3.640; *p* = 0.042 (0.766–0.985)
TI *	Therapist recognizes intentionality	0.957; F(9,5.820) = 5.250; *p* = 0.030 (0.786–0.990)
CP	Child proposes	1
TA	Therapist shares	1
SA	Social interplay/Shared activity	1
CX ***	Child’s withdrawal to interrupt the sharing	0.992; F(9,10) = 28.800; *p* < 0.001 (0.971–0.998)
TE	Therapist ends activity	1
CE **	Child ends the activity	0.975; F(9,10) = 8.700; *p* = 0.001 (0.908–0.994)
CD	Child’s signals of dysregulation	0.571; F(9,9.870) = 0.401; *p* = 0.907 (0.014–0.869)
TR	Therapist recognizes child’s emotional/activation state	0.667; F(9,9.960) = 0.556; *p* = 0.805 (0.138–0.904)
ENG	1—low engagement	0.943; F(9,5.91) = 3.9; *p* = 0.057
	2—medium engagement	0.741; F(9,6.56) = 0.738; *p* = 0.672
	3—high engagement	1

* *p* < 0.05; ** *p* < 0.01; *** *p* < 0.001.

**Table 2:** Behavioral descriptors.

Index	Description
N_TOT	Total number of behavioral events
N_SYNC	Total number of synchrony pairs
N_UDI	Total number of IUs (TP-CA; CP-TA; CI-TI)
P_CA ^a^	Proportional frequency of child’s acceptance
P_CX ^a^	Proportional frequency of child’s withdrawals
P_SA	Proportional frequency of shared activities
R_TPCA ^a^	Rate of therapist’s proposals over the total number of IUs
R_CITI ^a^	Rate of child’s intentionality signals over the total number of IUs
R_SYNC ^a^	Rate of synchrony code pairs over total code pairs
SR_TPCA	Rate of therapist’s proposals accepted by the child over the total number of therapist’s proposals
SR_CITI	Rate of child’s intentionality signals recognized by the therapist
R_SA ^a^	Rate of IUs that actually led to the initiation of a shared activity
R_SA_TPCA ^a^	Rate of therapist’s proposals that actually allowed for the initiation of a shared activity
LATENCY_TPCA ^a^ (s)	Mean latency between therapist’s proposals child’s acceptances
LATENCY_SA ^a^ (s)	Mean latency between IUs and the actual start of the shared activity
LATENCY_TWCA ^a^ (s)	Mean latency between therapist’s widenings and child’s acceptances
DURATION_SA ^a^ (s)	Mean duration of shared activities
R_CX ^a^	Rate of shared activities interrupted by child’s withdrawal
SRTWCA_SA ^a^	Rate of therapist’s widenings accepted by the child
N_BETWEEN_SA ^a^	Mean number of mid codes during shared activities (i.e., therapists widenings)
CPM ^a^	Codes per minute
SR_UDI ^a^	Rate of child’s acceptances over the total number of therapist’s proposals and widenings
ENG ^a^	Mean engagement level during shared activities

^a^ variables included in data modeling.

**Table 3:** Demographic and general statistics.

	T0 Mean (SD) [Range]	T1 Mean (SD) [Range]
Chronological age (months)	38.250 (9.988) (23–56)	53.583 (12.233) (33–75)
Mental age (months)	26.500 (7.223) (14–45)	39.33 (11.224) (18–63)
Socioeconomic Status (SES)	35.833 (13.230) (12.500–59.500)	
Time between T0 and T1 assessments (months)	14.333 (3.886) (8–23)	
Coding time (seconds)	1270 (109) (1184–1642)	1313 (122) (1133–1606)
	V = 98; *p* = 0.143; r^2^ = 0.303; BF = 0.408	

**Table 4:** Behavioral annotations and proportional frequencies.

Frequency Distributions			
Code	T0 Mean (SD) [Range]	T1 Mean (SD) [Range]	
Total * (TOT)	94.250 (27.227) (46–154)	74.708 (22.383) (35–123)	*t*(23) = 2.669; *p* = 0.014; *R*^2^ = 0.236; BF = 3.717
Proportional frequencies			
T. Proposes (TP)	0.123 (0.055) (0.022–0.222)	0.115 (0.057) (0.017–0.243)	*t*(23) = 0.687; *p* = 0.499; *R*^2^ = 0.020; BF = 0.266
T. Widens (TW)	0.253 (0.084) (0.126–0.391)	0.278 (0.094) (0.094–0.470)	*t*(23) = −1.027; *p* = 0.315; *R*^2^ = 0.043; BF = 0.344
C. Accepts ** (CA)	0.240 (0.061) (0.117–0.359)	0.299 (0.064) (0.186–0.424)	*t*(23) = −4.059; *p* < 0.001; *R*^2^ = 0.417; BF = 66.245
C. Rejects (CR)	0.021 (0.018) (0–0.067)	0.010 (0.015) (0–0.043)	-
C. Intentionality (CI)	0.074 (0.045) (0–0.181)	0.054 (0.039) (0–0.172)	*t*(23) = 1.764; *p* = 0.091; *R*^2^ = 0.119; BF = 0.819
T. Intentionality (TI)	0.070 (0.040) (0–0.149)	0.049 (0.036) (0–0.156)	*t*(23) = 1.940; *p* = 0.065; *R*^2^ = 0.141;BF = 1.063
C. Proposes (CP)	0.011 (0.015) (0–0.050)	0.014 (0.023) (0–0.105)	-
T. Accepts (TA)	0.011 (0.015) (0–0.050)	0.013 (0.023) (0–0.105)	-
C. Dysregulation (CD)	0.032 (0.043) (0–0.167)	0.021 (0.029) (0–0.110)	-
T. Recognizes (TR)	0.018 (0.024) (0–0.081)	0.016 (0.023) (0–0.090)	-
Shared Activity (SA)	0.074 (0.018) (0.029–0.097)	0.065 (0.022) (0.030–0.115)	*t*(23) = 1.794; *p* = 0.086; *R*^2^ = 0.123; BF = 0.855
C. Withdrawal * (CX)	0.060 (0.025) (0.011–0.097)	0.039 (0.026) (0–0.096)	*t*(23) = 3.305; *p* = 0.003; *R*^2^ = 0.322; BF = 13.142
C. Ends (CE)	0.005 (0.012) (0–0.045)	0.010 (0.012) (0–0.035)	-
T. Ends (TE)	0.009 (0.009) (0–0.031)	0.016 (0.021) (0–0.093)	-

* *p* < 0.05; ** *p* < 0.01.

**Table 5:** Changes in behavioral descriptors and developmental variables between T0 and T1.

Longitudinal Changes			
	T0 Mean (SD) [Range]	T1 Mean (SD) [Range]	Statistics
Behavioral Descriptors			
N_TOT *	94.250 (27.227) (46–154)	74.708 (22.383) (35–123)	*t*(23) = 2.669; *p* = 0.014; *R*^2^ = 0.236; BF = 3.717
N_SYNC	31.083 (90.12) (18–52)	27.958 (8.312) (11–44)	*t*(23) = 1.406; *p* = 0.173; *R*^2^ = 0.08; BF = 0.512
N_UDI	14.500 (6.228) (6–31)	10.792 (5.283) (1–19)	*t*(23) = 1.875; *p* = 0.074; *R*^2^ = 0.133; BF = 0.963
P_CA ***	0.240 (0.061) (0.117–0.359)	0.299 (0.064) (0.186–0.424)	*t*(23) = −4.059; *p* < 0.001; *R*^2^ = 0.417; BF = 66.245
P_CX **	0.060 (0.025) (0.011–0.097)	0.039 (0.026) (0–0.096)	*t*(23) = 3.305; *p* = 0.003; *R*^2^ = 0.322; BF = 13.142
P_SA	0.074 (0.018) (0.030–0.115)	0.065 (0.022) (0.030–0.115)	*t*(23) = 1.794; *p* = 0.086; *R*^2^ = 0.123; BF = 0.855
R_TPCA *	0.504 (0.249) (0.037–1)	0.616 (0.225) (0.143–1)	*t*(23) = −2.147; *p* = 0.043; *R*^2^ = 0.167; BF = 1.478
R_CITI *	0.424 (0.213) (0–0.815)	0.311 (0.185) (0–0.571)	*t*(23) = 2.355; *p* = 0.027; *R*^2^ = 0.194; BF = 2.102
R_SYNC ***	0.715 (0.092) (0.469–0.849)	0.836 (0.088) (0.600–0.967)	*t*(23) = −4.140; *p* < 0.001; *R*^2^ = 0.427; BF = 79.093
SR_TPCA **	0.618 (0.178) (0.250–1)	0.791 (0.174) (0.333–1)	*t*(23) = −3.483; *p* = 0.002; *R*^2^ = 0.345; BF = 19.07
SR_CITI	0.919 (0.125) (0.600–1)	0.878 (0.246) (0–1)	V = 77; *p* = 0.660; *R*^2^ = 0.069; BF = 0.272
R_SA	0.488 (0.163) (0.194–0.833)	0.506 (0.227) (0.211–1)	*t*(23) = −0.301; *p* = 0.766; *R*^2^ = 0.004; BF = 0.224
R_SA_TPCA	0.540 (0.282) (0–1)	0.538 (0.308) (0–1)	*t*(23) = 0.028; *p* = 0.978; *R*^2^ < 0.001; BF = 0.215
LATENCY_TPCA	2.747 (1.292) (0.280–6.091)	2.158 (1.114) (0.300–6.037)	*t*(23) = 1.786; *p* = 0.087; *R*^2^ = 0.122; BF = 0.846
LATENCY_SA	12.536 (5.491) (4.143–26.500)	14.234 (10.072) (2.33–46)	*t*(23) = −0.716; *p* = 0.481; *R*^2^ = 0.02; BF = 0.271
LATENCY_TWCA *	2.817 (0.854) (1.593–4.357)	2.291 (0.987) (0.712–4.522)	*t*(23) = 2.162; *p* = 0.041; *R*^2^ = 0.169; BF = 1.513
DURATION_SA *	144.328 (98.640) (47.533–523)	233.476 (140.148) (109–645)	V = 64; *p* = 0.013; *R*^2^ = 0.502; BF = 2.415
R_CX *	0.795 (0.237) (0.167–1)	0.584 (0.277) (0–1)	V = 205; *p* = 0.011; *R*^2^ = 0.540; BF = 5.336
SRTWCA_SA ***	0.572 (0.251) (0–1)	0.776 (0.185) (0.250–1)	*t*(23) = −3.982; *p* < 0.001; *R*^2^ = 0.408; BF = 55.914
N_BETWEEN_SA *	5.338 (4.447) (0.571–21.750)	8.145 (6.322) (2–28)	V = 74; *p* = 0.029; *R*^2^ = 0.44; BF = 1.857
CPM **	4.464 (1.276) (2.319–6.591)	3.421 (0.966) (1.307–5.390)	*t*(23) = 0.321; *p* = 0.004; *R*^2 =^ 0.309; BF = 10.726
SR_UDI ***	0.635 (0.114) (0.383–0.791)	0.767 (0.141) (0.419–0.944)	*t*(23) = −3.788; *p* < 0.001; *R*^2^ = 0.384; BF = 36.647
ENG ***	1.117 (0.170) (1–1.500)	1.694 (0.297) (1–2)	V = 0; *p* < 0.001; *R*^2^ = 0.872; BF > 100

* *p* < 0.05; ** *p* < 0.01; *** *p* < 0.001.

**Table 6:** Longitudinal changes in developmental outcomes and symptom severity.

Longitudinal Changes			
	T0 Mean (SD) [Range]	T1 Mean (SD) [Range]	Statistics
Developmental outcomes and symptoms severity			
Language Quotient *	55.417 (26.090) (25–120)	71.667 (34.949) (21–35)	V = 60.5; *p* = 0.0191; R^2^ = 0.473; BF = 7.637
rMC (mental age/chronological age) **	0.718 (0.199) (0.375–1.304)	0.782(0.276) (0.378–1.576)	*t*(23) = −2.862; *p* = 0.009; *R*^2^ = 0.263
Ados-2 Social Affect (SA) **	12.333 (3.319) (6–19)	10.458 (2.978) (6–16)	*t*(23) = 2.828; *p* = 0.010; *R*^2^ = 0.258; BF = 5.041
Ados-2 Restricted Repetitive Behaviors (RRB)	3.917 (1.640) (1–7)	3.667 (1.711) (1–7)	*t*(23) = 0.655; *p* = 0.519; *R*^2^ = 0.018; BF = 0.261

* *p* < 0.05; ** *p* < 0.01.

## Data Availability

The code is available under request to the corresponding author. The data that support the findings of this study are available upon reasonable request from the corresponding author. The data are not publicly available due to privacy reasons.
